# A Cross-Sectional Study of Dermatoses in Postmenopausal Patients at an Urban Tertiary Healthcare Center

**DOI:** 10.7759/cureus.80247

**Published:** 2025-03-08

**Authors:** C M Janani Sree, Sudha Rangarajan, Sai Preethi P, Adikrishnan S

**Affiliations:** 1 Dermatology, Sri Ramachandra Institute of Higher Education and Research, Chennai, IND

**Keywords:** atrophic vaginitis, estrogen deficiency, menopause, paraneoplastic dermatoses, photoaging

## Abstract

Background and objective

Menopause is a significant phase that marks the end of the reproductive years in women and heralds various physiological, hormonal, and psychological changes. There is scarce research on dermatoses in menopause, particularly in developing countries, where many cases go unreported or undiagnosed due to limited healthcare access and awareness. The study aimed to analyze the clinical pattern of dermatoses in postmenopausal women.

Methods

We conducted a cross-sectional study involving 200 cases of postmenopausal women with dermatoses in any area of the body treated at the Dermatology OPD, Sri Ramachandra Institute of Higher Education and Research (SRIHER) for a period of two years (July 2022-July 2024). A semi-structured questionnaire was circulated, which sought to document data on age, presenting complaints, associated symptoms suggestive of any underlying autoimmune, connective tissue disorder or malignancy, personal and family H/O diabetes mellitus, hypertension, endocrine disorders, cardiovascular diseases, dietary and menstrual history, socioeconomic history, sanitation history, and obstetric history.

Results

The study included a total of 200 postmenopausal patients who were evaluated for cutaneous, hair, nail, and oral cavity changes. The mean age of the participants was 58.99 ±9.12 years. The age at which menopause started was 40-50 years for most participants (n=127, 63.5%). Occupation-wise, most participants were housewives (n=178, 85.5%). Wrinkles, observed in 149 patients (74.5%), were the common physiological change noted. Of the pathological changes, fungal infections like tinea (n=29, 14.5%) were the most common. Among eczematous conditions, asteatotic eczema, observed in nine patients (4.5%), was the most common; as for papulosquamous disorders, psoriasis (n=22, 11%) was the most frequent condition. Seborrheic keratosis (n=43, 21.5%) was the most frequent benign tumor; regarding psychocutaneous disorders, lichen simplex chronicus (n=3, 1.5%) was the most commonly encountered condition.

Conclusions

Postmenopausal dermatoses represent a significant but underrecognized issue related to women's health in India. This study highlights the prevalence of conditions like atrophic vaginitis, lichen planus, and fungal infections in our postmenopausal population.

## Introduction

Menopause is a significant phase that marks the end of reproductive years in women and brings about various physiological, hormonal, and psychological changes [1]. There is limited research on dermatoses in postmenopausal women, especially in developing countries, where many cases go unreported or undiagnosed due to limited healthcare access and awareness [2]. The increasing life expectancy in our country, attributed to advancements in healthcare and economic improvements, underscores the importance of understanding and addressing menopausal issues [3]. As women now live longer, with an average expectancy of life of 68 years (which is expected to increase to 73 by 2021), dermatologists and healthcare providers need to be well-versed in the dermatological manifestations of menopause [4].

Menopause is defined as the permanent termination of menstruation, typically marked to have occurred after 12 successive months without a menstrual period due to declining ovarian follicular activity and decreasing estrogen levels [1]. This hormonal transition leads to a cascade of changes, encompassing hormonal shifts, physiological alterations, psychological adjustments, and sexual changes. The average age of menopause in India is about 46 years [5,6].

Dermatological conditions associated with menopause

These can be broadly classified into several categories

Physiological Changes

these encompass alterations in breast tissue, uterus, vagina, external genitalia, lower urinary tract, and hair growth patterns. The glandular tissue of the breast decreases, replaced by fibrous tissue. The uterus and vaginal muscles atrophy, external genitalia experiences atrophy of vulval fat, and the lower urinary tract may experience atrophic changes leading to an increased risk of prolapse and urinary infections [4].

Dermatoses Associated With Estrogen Deficiency

Its deficiency during menopause can lead to conditions such as atrophic vaginitis, vulvar lichen sclerosus, hirsutism, and others [4].

Non-specific Dermatoses

These are dermatological conditions that can occur during menopause but are not directly caused by hormonal changes, such as bullous pemphigoid, psoriasis, lichen planus, infections like dermatophytosis, and more [4].

Dermatoses Associated With Hormone Replacement Therapy (HRT)

Some women choose to undergo HRT to alleviate menopausal symptoms. However, HRT can be an etiological cause for certain dermatological issues such as melasma, darkening of nevi, and acanthosis nigricans [4].

The aging process itself contributes significantly to dermatological changes. Extrinsic aging occurs due to environmental factors like sun exposure and poor nutrition, while intrinsic aging is driven by genetic and biological factors such as telomere shortening. In postmenopausal women, there is a decrease in collagen, mast cells, fibroblasts, and blood vessels in the skin, leading to thinning, wrinkles, dryness, and fragility [7,8]. The study aims to examine the clinical pattern of dermatoses in postmenopausal women attending the OPD at the Dermatology Department of Sri Ramachandra Institute of Higher Education And Research (SRIHER).

## Materials and methods

The study was conducted in the Department of Dermatology, Venereology, and Leprosy at SRIHER, Porur, Chennai, India. It was a cross-sectional study carried out over two years, from July 2022 to July 2024. A sample size of 200 postmenopausal patients attending the Dermatology OPD at SRIHER was determined based on a level of significance of 5% and a precision of 5%.

The study population included all postmenopausal women with dermatoses affecting any area of the body who attended the Dermatology OPD at SRIHER during the study period. Subjects who met the eligibility criteria were invited to participate and were asked to provide written informed consent in their native language for convenience.

The inclusion criteria comprised all postmenopausal women with dermatoses who consented to participate in the study. The exclusion criteria included patients who were unwilling to provide written consent, women who had not yet attained menopause, severely immunocompromised patients due to any cause, and post-hysterectomy patients.

This cross-sectional study involved administering proforma to the participants, which collected data on age, presenting complaints, associated symptoms suggestive of underlying autoimmune, connective tissue disorders, or malignancies, as well as personal and family histories of diabetes mellitus, hypertension, endocrine disorders, cardiovascular diseases, dietary and menstrual history, socioeconomic background, sanitation, and obstetric history. The details were collected to gather knowledge on the lifestyle and demography of the patient population, which could be a factor in the etiology of dermatoses. The proforma was created based on our understanding of what could contribute to the causation of both physiological and pathological changes in postmenopausal women.

Written informed consent was obtained from all participants after explaining the objectives of the study, potential risks and benefits, and the voluntary nature of their participation. Each participant underwent a detailed general and dermatological examination. Specific investigations, including KOH smear, Wood’s lamp examination, Tzanck smear, and skin biopsy, were performed as required based on clinical indications.

The results were analyzed using SPSS Statistics Version 27.0 (IBM Corp., Armonk, NY).

## Results

Among the 200 study participants, the majority were aged 51-60 years (n=90, 45%), followed by those aged 61-70 years (n=56, 28%), 41-50 years (n=34, 17%), 71-80 years (n=12, 6%), and 81-90 years (n=8, 4%). The age at which menopause started was 40-50 years for most participants (n=127, 63.5%), and it was 51-60 years for the rest (36.5%). Regarding occupation, most participants were unskilled workers (89%), while 10.5% were skilled workers, and only 0.5% were professionals. Physiological skin changes were commonly observed, with wrinkles (74.5%) and xerosis (65.5%) being the most frequent, followed by idiopathic guttate hypomelanosis (IGH, 26.5%), senile comedones (12%), senile lentigines (5.5%), and atrophic vaginitis (2.5%) (Table 1).

**Table 1:** Demographic characteristic of the study participants (N=200) IGH: idiopathic guttate hypomelanosis

Sl no.	Variable	Frequency	Percentage
1	Age group, years		
	41-50	34	17
	51-60	90	45
	61-70	56	28
	71-80	12	6
	81-90	8	4
2	Age at menopause, years		
	40-50	127	63.5
	51-60	73	36.5
3	Occupation		
	Professional	1	0.5
	Skilled	21	10.5
	Unskilled	178	89
4	Physiological skin changes		
	Xerosis	131	65.5
	Wrinkles	149	74.5
	IGH	53	26.5
	Senile comedones	24	12
	Senile lentigines	11	5.5
	Atrophic vaginitis	5	2.5

Fungal infections were the most common, with tinea corporis reported in 14.5%, onychomycosis in 1.5%, and intertrigo in 2.5%. Bacterial and mycobacterial infections were less frequent, with erythrasma, ecthyma, folliculitis, cellulitis, secondary syphilis, and tuberculosis verrucosa cutis accounting for 1.5%, 0.5%, 0.5%, 1.0%, 0.5%, and 0.5% respectively. Viral infections, including herpes zoster and verruca vulgaris, were noted in 1.5% and 0.5%, respectively. Parasitic infections such as scabies, cutaneous larva migrans, and filariasis were rare, each seen in 0.5% of the participants (Table 2).

**Table 2:** Distribution of types of infection among the study participants (N=200)

Sl no.	Types of infection	Frequency	Percentage
1	Fungal		
	Tinea corporis	29	14.5
	Onychomycosis	3	1.5
	Intertrigo	5	2.5
2	Bacterial and mycobacterial		
	Erythrasma	3	1.5
	Ecthyma	1	0.5
	Folliculitis	1	0.5
	Cellulitis	2	1
	Secondary syphilis	1	0.5
	Tuberculosis verrucosa cutis	1	0.5
3	Viral		
	Herpes zoster	3	1.5
	Verruca vulgaris	1	0.5
4	Parasitic		
	Scabies	1	0.5
	Cutaneous larva migrans	1	0.5
	Filariasis	1	0.5

Eczematous conditions were observed in various forms, with asteatotic eczema being the most common (4.5%), followed by chronic eczema (2.5%) and irritant contact dermatitis (1.0%). Papulosquamous disorders such as psoriasis (11%) and lichen planus (6.5%) were also noted. Among types of psoriasis, palmoplantar psoriasis (7%) was the most frequent. Pigmentary dermatoses, including melasma (17.5%), vitiligo (4%), and lichen planus pigmentosus (0.5%), were documented in varying proportions (Table 3).

**Table 3:** Distribution of pathological skin changes among the study participants (N=200)

Sl no.	Types of infection	Frequency	Percentage
1	Eczematous conditions		
	Chronic eczema	5	2.5
	Hand eczema	1	0.5
	MR eczema	2	1
	Irritant contact dermatitis	2	1
	Allergic contact dermatitis	4	2
	Photoallergic contact dermatitis	1	0.5
	Airborne contact dermatitis	1	0.5
	Frictional dermatitis	1	0.5
	Drawstring dermatitis	1	0.5
	Asteatotic eczema	9	4.5
2	Papulosquamous disorders		
	Psoriasis	22	11
	Lichen planus	13	6.5
3	Types of psoriasis		
	Palmoplantar psoriasis	14	7
	Psoriasis vulgaris	5	2.5
	Sebopsoriasis	3	1.5
4	Pigmentary dermatoses		
	Melasma	35	17.5
	Vitiligo	8	4
	Lichen planus pigmentosus	1	0.5

Benign tumors were the most common, with seborrheic keratosis (21.5%) and acrochordons (16%) being the most frequent, followed by dermatosis papulosa nigra (DPN) (12.5%), cherry angiomas (5%), and a single case of glomus tumor (0.5%). Pre-malignant tumors such as Bowen's disease, actinic granuloma, and Jessner's lymphocytic infiltrate were rare, each accounting for 0.5-1%. Malignant tumors like angiosarcoma and basal cell carcinoma were also rare, with one case (0.5%) of each reported (Table 4).

**Table 4:** Distribution of tumors of skin among the study participants (N=200) DPN: dermatosis papulosa nigra

Sl no.	Types of tumors	Frequency	Percentage
1	Benign		
	Seborrhoeic keratosis	43	21.5
	DPN	25	12.5
	Cherry angiomas	10	5
	Acrochordons	32	16
	Glomus tumor	1	0.5
2	Premalignant tumors		
	Bowen's disease	2	1
	Actinic granuloma	1	0.5
	Jessners lymphocytic infiltrate	1	0.5
3	Malignant tumors		
	Angiosarcoma	1	0.5
	Basal cell carcinoma	1	0.5

Pemphigus vulgaris was the most frequent autoimmune disorder (n=3, 1.5%, followed by bullous pemphigoid (n=2, 1.0%). Several other autoimmune disorders were present but were less common, each affecting 0.5% of the population (n=1 each). These include pemphigus foliaceous, systemic lupus erythematosus (SLE), and dermatomyositis (Table 5).

**Table 5:** Distribution of autoimmune disorders among the study participants (N=200) SLE: systemic lupus erythematosus

Sl no.	Autoimmune disorders	Frequency	Percentage
1	Pemphigus vulgaris	3	1.5
2	Bullous pemphigoid	2	1
3	Pemphigus foliaceous	1	0.5
4	SLE	1	0.5
5	Dermatomyositis	1	0.5

Among miscellaneous dermatoses, lichen amyloidosis was the most common (n=13, 6.5%). This was followed by polymorphous light eruption (PMLE) and miliaria rubra, each affecting 3.0% of the population (n=6 each). Other reported conditions included senile pruritus and acquired ichthyosis, each affecting 1.5% of the population (n=3 each) (Table 6).

**Table 6:** Distribution of miscellaneous dermatoses among the study participants (N=200) CKD: chronic kidney disease; PMLE: polymorphous light eruption

Sl no.	Miscellaneous dermatoses	Frequency	Percentage
1	Lichen amyloidosis	13	6.5
2	Senile pruritus	3	1.5
3	Colloid milia	2	1
4	Acquired ichthyosis	3	1.5
5	Anetoderma	1	0.5
6	Kyrle's disease secondary to CKD	1	0.5
7	Lymphedema with lymphangiectasia	1	0.5
8	Vulval lymphedema	1	0.5
9	Acute urticaria	2	1
10	Chronic urticaria	1	0.5
11	Corn foot/callosity	2	1
12	Lseta	2	1
13	PMLE	6	3
14	Lipodermatosclerosis	1	0.5
15	Morphea	1	0.5
16	Miliaria rubra	6	3
17	Dowling degos	1	0.5
18	Trichothiodystrophy	1	0.5
19	Keloid	1	0.5
20	Bartholin cyst	1	0.5
21	Intradermal nevus	1	0.5
22	Fixed drug eruption	1	0.5
23	Papular urticaria	2	1

Lichen simplex chronicus was the most frequent psychocutaneous disorder (n=3, 1.5%). Delusional parasitosis was the least common, affecting 0.5% of the population (n=1) (Table 7).

**Table 7:** Distribution of psychocutaneous disorders among the study participants (N=200)

Sl no.	Psychocutaneous disorders	Frequency	Percentage
1	Lichen simplex chronicus	3	1.5
2	Delusional parasitosis	1	0.5

Hair changes were common in the cohort, with canities (76%) being the most frequently observed, followed by telogen effluvium (13%), seborrhoeic dermatitis (3%), and scalp psoriasis (1.5%). Nail changes encompassed varied presentations, with longitudinal ridges and brittle nails being the most common (18.5% each), followed by onycholysis (18.5%), chronic paronychia (4.5%), nail discoloration (6%), and other changes like pitting, subungual hyperkeratosis, and koilonychia occurring less frequently. Oral cavity changes included dental caries (13%) as the most common, with other findings such as poor oral hygiene (3%), oral lichen planus (1.5%), candidiasis (0.5%), morsicatio buccarum (2%), aphthous ulcers (0.5%), and mucosal pigmentation (0.5%) being less frequent (Table 8).

**Table 8:** Distribution of hair changes, nail changes, and oral cavity changes among the study participants (N=200)

Sl no.	Variable	Frequency	Percentage
1	Hair changes		
	Canities	152	76
	Telogen effluvium	26	13
	Seborrhoeic dermatitis	6	3
	Scalp psoriasis	3	1.5
2	Nail changes		
	Pitting	20	10
	Subungual hyperkeratosis	6	3
	Brittle nails	6	3
	Onycholysis of fingernails	37	18.5
	Koilonychia	8	4
	Nail plate thickening	4	2
	Nail discoloration	12	6
	Dystrophy of nails	7	3.5
	Chronic paronychia	9	4.5
	Longitudinal ridges	37	18.5
3	Oral cavity changes		
	Caries	26	13
	Oral lichen planus	3	1.5
	Candidiasis	1	0.5
	Morsicatio buccarum	4	2
	Aphthous ulcers	1	0.5
	Pigmentation of mucosa	1	0.5
	Poor oral hygiene	6	3

## Discussion

The mean age of the study participants was 58.99 ±9.12 years (range: 42-89 years). In a study by Pariath et al. [9], most patients belonged to the age group of 61-70 (38.67%) with a mean age of 61.52 years. In a study by Pallavi et al. [10], the mean age was 58.4 ± 5.1 years, and there were 88 (39.5%) women in the age group of 51-60 years. In Shah et al.'s study [11], the ages of patients ranged from 42-65 years with a mean age of 54.83 ±5.73 years, and the age group of 56-60 years (26.9%) was the most prevalent. The mean age at which menopause started was 49.37 ±4.22 years (range: 40-56 years). The majority of the population was in the age range of 40-50 years at the start of menopause (n=127, 63.5%). In the study by Pariath et al. [9], participants largely belonged to the age group of 40-50 years (57.33%). In a study by Shah et al. [11], most patients belonged to the age group of 41-45 years (54.6%). In Pallavi et al.'s study [10], most of the patients belonged to the age group of 41-50 years (52.9%). Vatankhah et al. [12] reported a mean age of 48.63 ±5.37 regarding the start of menopause.

Our participants were predominantly housewives, constituting 85.5% of the total sample, which is significantly higher than the numbers reported by Shah et al. [11] (60.2%) and Pariath et al. [9] (54.67%). This indicates a more substantial domestic role among the women in our surveyed population. Conversely, Shah et al. [11] and Pariath et al. [9] reported that a significant portion of their populations were employed in sedentary jobs (36.6% and 42.67%, respectively), while our study had a much lower representation in similar categories, such as shopkeepers (5.0%) and office workers (0.5%).

In our study, wrinkles were the most frequent physiological change, affecting 74.5% of the participants: significantly higher than the 43.43% reported by Shah et al. [11] and comparable to the 48% reported by Pariath et al. [9]; however, it was lower than those reported by Bravo et al. [13] (43%), which could be attributed to UV damage on skin structure, function and appearance [14]. Xerosis, or dry skin, was seen in 65.5% of the population, which was notably higher than the 29.14% reported by Shah et al. [11]. IGH was seen in 26.5% of our sample, while senile comedones were present in 12% of the population. Senile lentigines were seen in 5.5% of our population, and atrophic vaginitis in 2.5%. Pariath et al. [9] reported a high prevalence of skin sagging: in 97.33% of their patients.

Pathological skin changes

In our study, various eczematous conditions were observed, with asteatotic eczema being the most common (4.5%), followed by chronic eczema (2.5%), allergic contact dermatitis (2.0%), MR eczema (1.0%), irritant contact dermatitis (1.0%), and other less common types (0.5% each). Pariath et al. [9] reported an overall eczema prevalence of 8.16%, without any specific mention of asteatotic eczema. Shah et al. [11] documented a lower prevalence of asteatotic eczema (2.57%), while Pallavi et al. [10] reported 3.2% for asteatotic eczema, 2.7% for allergic contact dermatitis, and 1.6% for irritant contact dermatitis. Our findings indicate a higher prevalence of asteatotic eczema than Shah et al. [11] and Pallavi et al. [10] but a lower overall eczema prevalence compared to Pariath et al. [9].

Papulosquamous disorders in our study included psoriasis (11%) and lichen planus (6.5%). This aligns with Pariath et al. [9], who reported psoriasis in 9.18% of their cohort but had a higher prevalence of lichen planus (11.22%). Shah et al. [11] did not specifically mention psoriasis, while Pallavi et al. [10] reported lower prevalences of psoriasis (3.2%) and lichen planus (2.7%). The incidence of psoriasis in our study is comparable to Pariath et al. [9] but higher than in Pallavi et al. [10], whereas lichen planus is less frequent than in Pariath et al. [9] but more common than in Pallavi et al. [10].

Among infective conditions in our study, fungal infections were the most prevalent (18.5%), followed by bacterial (4.5%), viral (2.0%), and parasitic infections (1.5%). Pariath et al. [9] reported a comparable prevalence of superficial dermatophytosis (16.33%) and lower bacterial infections (2.04%), but a higher incidence of herpes zoster (3.06%). Shah et al. [11] documented a higher prevalence of tinea cruris (26.06%) and vulvovaginal candidiasis (16.97%), while Pallavi et al. [10] reported superficial dermatophytosis at 21.0% and herpes zoster and postherpetic neuralgia at 4.8%. Fungal infections in our study were consistent with Pariath et al. [9] and Pallavi et al. [10] but lower than Shah et al. [11].

Benign tumours of the skin in our study included seborrheic keratosis (21.5%), acrochordons (16%), dermatosis papulosa nigra (12.5%), cherry angiomas (5%), and glomus tumour (0.5%). Compared to Pariath et al. [9] who reported seborrheic keratosis at 3.06%, our study showed a much higher prevalence. Shah et al. [11] reported seborrheic keratosis at 7.14%, acrochordons at 16.86%, dermatosis papulosa nigra at 10.86%, and cherry angiomas at 0.29%, while Pallavi et al. [10] documented much lower frequencies of seborrheic keratosis (1.1%) and acrochordons (2.2%). Our study had a notably higher prevalence of seborrheic keratosis and cherry angiomas compared to other studies.

Bullous disorders in our study included pemphigus vulgaris (1.5%) and bullous pemphigoid (1.0%), which align closely with Pariath et al. [9] (pemphigus vulgaris, 1.02%) and Pallavi et al. [10] (pemphigus vulgaris, 1.6%; bullous pemphigoid, 1.1%). Miscellaneous conditions in our study were diverse, with lichen amyloidosis (6.5%) being the most common, followed by polymorphous light eruption (PMLE) (3.0%), miliaria rubra (3.0%), senile pruritus (1.5%), and acquired ichthyosis (1.5%). Other less common conditions, including hirsutism and melasma, were each reported at 0.5%. Pariath et al. [9] documented melasma at 7.14% and vitiligo at 4.08%, while Shah et al. [11] noted idiopathic guttate hypomelanosis (7.71%) and senile comedones (1.43%). Pallavi et al. [10] reported melasma (4.3%), vitiligo (2.7%), and hirsutism (1.1%). Our study demonstrates a higher prevalence of lichen amyloidosis and includes a broader spectrum of conditions not prominently mentioned in other studies.

Hair changes

In our study, canities was the most common hair change, affecting 76%. This is higher than the 26.28% reported by Shah et al. [11], and the 45.89% reported as depigmentation by Pariath et al. [9] Telogen effluvium affects 13% of our population, which is slightly higher than Shah et al. [11] 11.14% and Pallavi et al. [10] 32%, where diffuse hair loss is a major category. Pariath et al. [9] report a lower prevalence of diffuse hair loss at 4.10%. Seborrhoeic dermatitis affects 3% of our population. Scalp psoriasis affects 1.5% of our population. Pariath et al. [9] report female pattern hair loss as the most common condition, affecting 34.24%, while Shah et al. [11] and Pallavi et al. [10] report 34% and 46.7%, respectively. Alopecia areata is reported by Pariath et al. [9] (5.47%), Shah et al. [11] (2.28%), and Pallavi et al. [10] (2.7%). Male pattern hair loss is reported by Pallavi et al. [10] at 14.7% and by Pariath et al. [9] at 10.27%.

Other conditions, such as lichen planopilaris, were reported by Pallavi et al. [10] at 4%, and Shah et al. [11] included a category for others at 6.28%. Overall, our study shows a higher prevalence of canities and telogen effluvium compared to the other studies, while conditions like female pattern hair loss and alopecia areata were more prominently reported in the other studies. Canities or graying of hair is a chronological ageing process that occurs regardless of gender or race. The significance of including it in the study is that it's an observational study of all the changes noted in postmenopausal women, and since canities is one of the most common changes, it was included in the study.

Nail changes

In our study, the most prevalent nail change was onycholysis of fingernails and longitudinal ridges, affecting 18.5% of the population each. This contrasts with the findings of Pariath et al. [9], where onychoschizia was the most common condition, affecting 48.03% of their sample. Onychomycosis was reported as the most prevalent condition by Pallavi et al. [10], affecting 43.1% of their participants. Pitting was observed in 10% of our population, which is higher than the 2.94% reported by Pariath et al. [9] and the 8.6% reported by Pallavi et al. [10]. Nail discolouration affects 6% of our population. Chronic paronychia affects 4.5% of our population, comparable to Pallavi et al. [10] 13.8% but higher than Pariath et al. [9] 0.98%. Koilonychia affects 4% of our population, which is higher than the 1.96% reported by Pariath et al. [9]. Subungual hyperkeratosis affects 3% of our population, compared to 0.98% in Pariath et al. [9] study and 5.2% in Pallavi et al. [10] study. Brittle nails affect 3% of our population, whereas onychorrhexis was reported by Pariath et al. [9] at 11.76% and by Pallavi et al. [10] at 13.8%, and our study showed 18.5%. Dystrophy of nails affects 3.5% of our population, similar to the 2.94% reported by Pariath et al. [9]. Our study shows a diverse range of nail conditions, with a higher prevalence of onycholysis and longitudinal ridging. 

Oral cavity changes

In our study, dental caries was the most frequent oral cavity-related issue, affecting 13%, whereas Shah et al. [11] and Pariath et al. [9] report a higher prevalence of toothache or periodontal disease at 21.96% and 10%, respectively. Pallavi et al. [10] do not specifically mention toothache but include other oral cavity dermatoses. Aphthous ulcers affect 14.63% of Shah et al. [11] cases, 6.25% in Pariath et al. [9] study, and 1.8% in Pallavi et al. [10] study. Lichen planus was reported by Shah et al. [11] at 18.29%, by Pariath et al. [9] at 13.75%, and by Pallavi et al. [11] at 4%. Our study reports poor oral hygiene in 3% of the population and several conditions like oral lichen planus (1.5%), candidiasis (0.5%), morsicatio buccarum (2.0%), aphthous ulcers (0.5%), and pigmentation of mucosa (0.5%). Pariath et al. [9] highlight burning mouth syndrome (42.5%) and dryness of the mouth (26.25%). Pallavi et al. [10] also report burning mouth syndrome (1.3%) and dryness of the mouth (2.2%), albeit at much lower frequencies. Our study presents a higher prevalence of dental caries.

Limitations

This study has a few limitations, such as its single-center design and its observational nature. Hence, we recommend multicentric studies to validate our findings among the broader population of postmenopausal women.

## Conclusions

Postmenopausal dermatoses represent a significant but under-recognized issue related to women's health in India. The fall in estrogen levels in menopause disrupts the delicate hormonal balance of the skin, leading to a diverse array of cutaneous manifestations. This study has highlighted the prevalence of conditions like atrophic vaginitis, lichen planus, and fungal infections in our postmenopausal population. Further research is warranted to explore the specific factors influencing the presentation and course of postmenopausal dermatoses in the Indian context.

## Appendices

Proforma employed in the study

1. Address:                                               Phone number: 

2. Age (in years):  

3. Occupation: 

4. Presenting complaints: 

PAST HISTORY:

H/O diabetes mellitus, asthma, thyroid disease, HTN, liver or renal disease, TB, rheumatoid arthritis

H/O any surgery or disease in the past

H/O radiotherapy or chemotherapy

DRUG HISTORY:

TREATMENT HISTORY:

PERSONAL HISTORY:

Dietary history

Bowel and bladder habits

H/O tobacco usage

FAMILY HISTORY:

Skin diseases

Atopy

Others

SOCIOECONOMIC STATUS:

House/old age home

No. of family members

Occupation/retired

Nature of work

No. of hours of sun exposure

EDUCATION HISTORY:

SANITATION:

Bathing habits

 Frequency of baths

 Use of scrubs

 Temperature of water

  Frequency/ bed linen of washing clothes

Water availability

      Sewage/waste disposal

      Use of mosquito repellents

      Coils

      Creams

      Net

VACCINATION HISTORY:

MENSTRUAL AND OBSTETRIC HISTORY:

A. H/O oligomenorrhoea/menorrhagia

B. H/O dysmenorrhea

C. H/O irregular menstrual cycles 

D. No: of menstrual cycles/year

E. Average length of cycles

F. Days of menstrual flow 

G. No. of children

H. Mode of delivery=Normal/LSCS

I. Age of menopause

J. H/O hysterectomy 

K. H/O hot flashes/dryness of skin/sagging of skin/

Weight gain/sweating/itching/bladder discomfort

 L. H/O vaginal dryness/vaginal discharge/sexual discomfort/

     Burning micturition

EXAMINATION

1) GENERAL EXAMINATION:

     Height

     Weight

     Respiratory rate

     Pulse rate

     BP

     Temperature

      Pallor

      Cyanosis

      Clubbing

      Icterus

      Oedema

      Lymphadenopathy

2) SYSTEMIC EXAMINATION:

     CVS

     RS

     Per abdomen

     CNS

3) PHYSIOLOGICAL SKIN CHANGES RELATED TO MENOPAUSE:

    A. Wrinkles-YES/NO

    B. Xerosis-YES/NO

    C. Acrochordon-YES/NO

    D. Dermatoheliosis-YES/NO

    E. Idiopathic guttate hypomelanosis-YES/NO

    F. Seborrhoeic keratosis-YES/NO

    G. Melasma-YES/NO

    H. Asteatotic eczema-YES/NO

    I. Keratoderma climatricum-YES/NO

    J. Senile comedones-YES/NO

    K. Xanthelesma-YES/NO

    L. Melanocytic naevi-YES/NO

4) DERMATOLOGICAL EXAMINATION:

     Onset of lesion-sudden/gradual

Description of lesion

Distribution/site of lesion

Progression of lesion

Any co-existing condition

Pathological changes

1. Pigmentation:

a) hyperpigmentation-YES/NO

b) hypopigmentation-YES/NO

c) depigmentation-YES/NO

d) purpuric lesion-YES/NO

2. Macule

    Patch

    Papule

    Plaque

    Pustule

    Vescicle

    Nodule

    Wheals

3. Bullae:

    Site

    Size

    Number

4. Scales

    Crusts

    Excoriation

    Erosions

    Fissures

    Scars

5. Ulcer

     Site

     Size

     Discharge

6. Erythema

EXAMINATION OF SCALP, HEAD, AND NECK

ORAL CAVITY:

HAIR CHANGES:

NAIL CHANGES:

A. Nail plate

B. Nail matrix

C. Nail bed

GENITAL EXAMINATION

INVESTIGATIONS

DIAGNOSIS

TREATMENT

## References

[1] McNeil MA, Merriam SB (2021). Menopause. Ann Intern Med.

[2] Kalhan M, Singhania K, Choudhary P, Verma S, Kaushal P, Singh T (2020). Prevalence of menopausal symptoms and its effect on quality of life among rural middle-aged women (40-60 years) of Haryana, India. Int J Appl Basic Med Res.

[3] Crimmins EM (2015). Lifespan and healthspan: past, present, and promise. Gerontologist.

[4] Nair PA (2014). Dermatosis associated with menopause. J Midlife Health.

[5] Meeta M, Digumarti L, Agarwal N, Vaze N, Shah R, Malik S (2020). Clinical practice guidelines on menopause: an executive summary and recommendations: Indian Menopause Society 2019-2020. J Midlife Health.

[6] Sullivan SD, Sarrel PM, Nelson LM (2016). Hormone replacement therapy in young women with primary ovarian insufficiency and early menopause. Fertil Steril.

[7] Ahuja M (2016). Age of menopause and determinants of menopause age: A PAN India survey by IMS. J Midlife Health.

[8] Ceylan B, Özerdoğan N (2015). Factors affecting age of onset of menopause and determination of quality of life in menopause. Turk J Obstet Gynecol.

[9] Pariath K, Nair PA (2019). A cross-sectional study on the dermatoses in postmenopausal patients at a rural-based tertiary health care center. Indian J Dermatol.

[10] Pallavi UK, Sinha R, Chandan Jaykar K, Sarkar S, Yasmeen T, Prasad D (2023). Dermatoses in postmenopausal women in a tertiary health care center of Bihar: a prospective cross-sectional study. Cureus.

[11] Shah B, Patvekar MA, Singh P, Deora MS, Alisha Alisha (2020). Pattern of dermatological involvement in early postmenopausal period: a cross-sectional study. Int J Res Dermatol.

[12] Vatankhah H, Khalili P, Vatanparast M, Ayoobi F, Esmaeili-Nadimi A, Jamali Z (2023). Prevalence of early and late menopause and its determinants in Rafsanjan cohort study. Sci Rep.

[13] Bravo B, Penedo L, Carvalho R (2024). Dermatological changes during menopause and HRT: what to expect?. Cosmetics.

[14] Zouboulis CC, Blume-Peytavi U, Kosmadaki M, Roó E, Vexiau-Robert D, Kerob D, Goldstein SR (2022). Skin, hair and beyond: the impact of menopause. Climacteric.

